# Forecasting incidence of hemorrhagic fever with renal syndrome in China using ARIMA model

**DOI:** 10.1186/1471-2334-11-218

**Published:** 2011-08-15

**Authors:** Qiyong Liu, Xiaodong Liu, Baofa Jiang, Weizhong Yang

**Affiliations:** 1 National Institute for Communicable Disease Control and Prevention, China CDC, Beijing, 102206, PR China; 2 State Key Laboratory for Infectious Diseases Prevention and Control, Beijing, 102206, PR China; 3 Department of Epidemiology and Health Statistics, School of Public Health, Shandong University, Jinan, 250012, Shandong Province, PR China; 4 Chinese Center for Disease Control and Prevention, Beijing, 102206, PR China

## Abstract

**Background:**

China is a country that is most seriously affected by hemorrhagic fever with renal syndrome (HFRS) with 90% of HFRS cases reported globally. At present, HFRS is getting worse with increasing cases and natural foci in China. Therefore, there is an urgent need for monitoring and predicting HFRS incidence to make the control of HFRS more effective. In this study, we applied a stochastic autoregressive integrated moving average (ARIMA) model with the objective of monitoring and short-term forecasting HFRS incidence in China.

**Methods:**

Chinese HFRS data from 1975 to 2008 were used to fit ARIMA model. Akaike Information Criterion (AIC) and Ljung-Box test were used to evaluate the constructed models. Subsequently, the fitted ARIMA model was applied to obtain the fitted HFRS incidence from 1978 to 2008 and contrast with corresponding observed values. To assess the validity of the proposed model, the mean absolute percentage error (MAPE) between the observed and fitted HFRS incidence (1978-2008) was calculated. Finally, the fitted ARIMA model was used to forecast the incidence of HFRS of the years 2009 to 2011. All analyses were performed using SAS9.1 with a significant level of *p *< 0.05.

**Results:**

The goodness-of-fit test of the optimum ARIMA (0,3,1) model showed non-significant autocorrelations in the residuals of the model (Ljung-Box Q statistic = 5.95,*P *= 0.3113). The fitted values made by ARIMA (0,3,1) model for years 1978-2008 closely followed the observed values for the same years, with a mean absolute percentage error (MAPE) of 12.20%. The forecast values from 2009 to 2011 were 0.69, 0.86, and 1.21per 100,000 population, respectively.

**Conclusion:**

ARIMA models applied to historical HFRS incidence data are an important tool for HFRS surveillance in China. This study shows that accurate forecasting of the HFRS incidence is possible using an ARIMA model. If predicted values from this study are accurate, China can expect a rise in HFRS incidence.

## Background

Hemorrhagic fever with renal syndrome (HFRS), or epidemic hemorrhagic fever (EHF) is an acute viral syndrome caused by infection with one of hantaviruses. HFRS is an important infectious disease in developing countries. In China, HFRS is caused mainly by 2 types of hantaviruses, Hantaan virus (HTNV) and Seoul virus (SEOV), each of which has coevolved with a distinct rodent host. HTNV is associated with *Apodemus agrarius*, whereas SEOV, which causes a less severe form of HFRS, is associated with *Rattus norvegicus *[1]. In hantavirus -endemic areas, HFRS is most common among farmers and others who may have close contact with excreta of infected rodents [2,3]. In mainland China, HFRS remains a serious public health problem with approximately 20,000-50,000 human cases reported annually, approximately 90% of the total cases worldwide [4-6]. Currently, HFRS is endemic in 28 of 31 provinces in mainland China [4,7].

In response to the spread of HFRS in China, the Chinese Center for Disease Control and Prevention designed a surveillance system for HFRS and created educational programs for the general public. However, the impact of control efforts remains difficult to measure due to the inherent complexities of HFRS as a disease: multiple viral strains with identified genetic polymorphisms, complex disease manifestation, diverse animal reservoirs, and multiple routes of transmission [8]. Infectious diseases have certain characteristic features that lead themselves to modeling, such as: speed of pathogen variation, accumulation of susceptible hosts, and environmental indices [9]. Thus, epidemic modeling and forecasting can be essential tools to prevent and control HFRS. Recently, statistical methods including linear regression [10-12], correlation coefficients [13], grey swing model [14], back propagation artificial neural network model [15] have been used for prediction of HFRS incidence. The variation of HFRS incidence, which is influenced and constrained by diversified factors, is characterized by tendency and randomicity. These statistical tools are inappropriate for analyzing the randomicity of HFRS. Autoregressive integrated moving average (ARIMA) models, which take into account changing trends, periodic changes, and random disturbances in time series, are very useful in modeling the temporal dependence structure of a time series. In epidemiology, ARIMA models have been successfully applied to predict the incidence of infectious diseases, such as influenza mortality [16], malaria incidence [17], as well as other infectious diseases [18,19]. This study aimed to develop a univariate time series model for the HFRS incidence; specifically, a stochastic ARIMA model, for short-term forecasting of HFRS incidence (per 100,000 population) in China.

## Methods

### Materials

Chinese HFRS incidence data from 1975 to 2008 was obtained from the Chinese Center for Disease Control and Prevention. All HFRS cases were initially diagnosed by clinical symptoms. Patient blood samples were also collected and sent to local Centers for Disease Control and Prevention (CDC) laboratories for serological confirmation. Finally, data were collected by case number according to the sampling results. There might be admission rate bias in the disease report, but this has been reduced as much as possible. In China, HFRS is a nationally notifiable disease and hospital physicians must report every case of HFRS to the local health authority within 12 hours. Local health authorities later report monthly HFRS case totals to higher the national level CDC for surveillance purposes. Due to mandatory reporting, it is believed that the degree of compliance in disease notification over the study period was consistent.

We used the Box-Jenkins approach to ARIMA (p, d, q) modeling of time series [20]. This model-building process is designed to take advantage of associations in the sequentially lagged relationships that usually exist in periodically collected data [21]. The following were the parameters selected when fitting the ARIMA model: *p*, the order of autoregression; *d*, the degree of difference; *q*, the order of moving average.

The annual data used in this study did not show seasonal pattern, so the series was differenced at the non-seasonal level to induce stationarity. Autocorrelation function (ACF) graph and Partial autocorrelation function (PACF) graph were used to identify the order of moving average (MA) and autoregressive (AR) terms included in the ARIMA model. Estimates of the model's parameters were obtained by the conditional least squares method. Diagnostic checking including residual analysis and the Akaike Information Criterion (AIC) was used to compare the goodness-of-fit among ARIMA models. The Ljung-Box test was used to measure the ACF of the residuals. In addition, we used the mean absolute percentage error (MAPE) and fitting effect diagram to assess forecast accuracy.

, where *x_t _*and denote observed and fitted values at time point t. The MAPE value was calculated based on observed values and fitted values from 1978 to 2008. A lower MAPE value indicates a better fit of the data. Finally, the fitted ARIMA model was used for short-term forecasting of HFRS incidence for years 2009 to 2011. All analyses were performed using SAS9.1 with a significant level of *p *< 0.05.

### Ethical review

The present study was reviewed by the research institutional review board of Shandong University and the China CDC, and found that utilization of disease surveillance and meteorological data did not require oversight by an ethics committee.

## Results

### Temporal analysis

**Figure 1:**
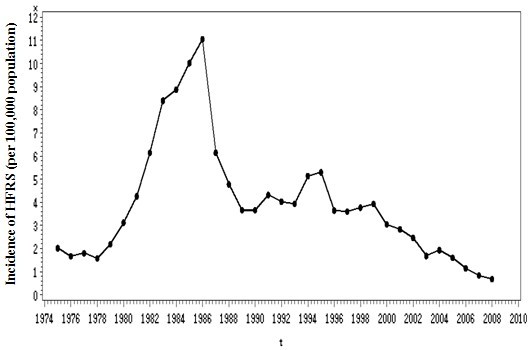
**The incidence of HFRS in China from 1975 to 2008**.

From 1975 to 1986, the HFRS incidence in China rose regularly with a peak in 1986 of 11.06 cases per one hundred thousand population. After 1986, the incidence descended sharply with a dramatic fluctuation until 2008 (Figure 1). The lowest incidence could be seen in 2008, 0.68 per one hundred thousand.

### Model identification

**Figure 2:**
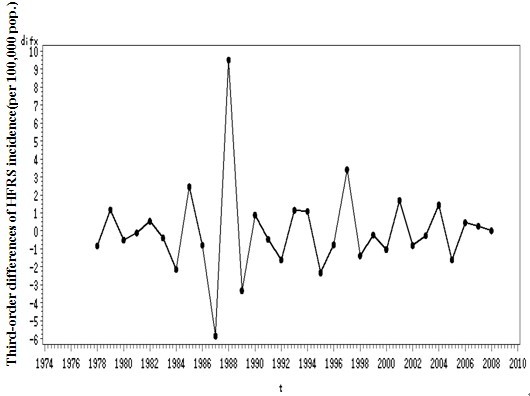
**Third-order differences of HFRS incidence(per 100,000 pop.)**.

**Figure 3:**
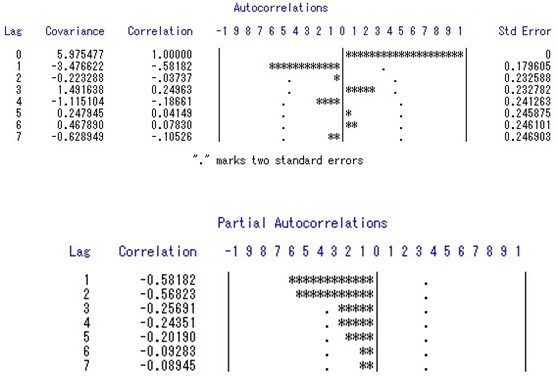
**Autocorrelation(acf, top) and partial autocorrelation(pacf, bottom) functions of third-order differences HFRS incidence**. Dotted line: 95% confidence intervals.

According Figure 1, the series showed a non-stationary mean, so we stabilized the mean of HFRS incidence by taking both second and third order differences. All further statistical procedures were performed on the transformed HFRS incidence. Based on the distribution characteristics, we conducted five models, ARIMA(0, 2, 1), ARIMA(1,2,1), ARIMA(0,3,1), ARIMA(1, 3, 1), and ARIMA(2, 3, 1). Of all the models tested, the ARIMA(0,3,1) model was the best fit for the data (Table 1). The transformation series by taking third-order differences is shown in Figure 2. The plots of ACF and PACF (Figure 3) described the temporal dependence structure in HFRS incidence. The slow decay in the PACF, associated with a ACF cutoff at lag 1 suggested a MA(q = 1).

**Table 1:** Comparisons of tested models

Model	Ljung-Box Q statistic	*P *value	AIC
**ARIMA(0,2,1)**	**5.78**	**0.3869**	**117.7200**
**ARIMA(1,2,1)**	**7.97**	**0.0488**	**119.6435**
**ARIMA(0,3,1)**	**5.95**	**0.3113**	**116.7075**
**ARIMA(1,3,1)**	**8.46**	**0.0453**	**118.6647**
**ARIMA(2,3,1)**	**9.33**	**0.0260**	**120.1425**

### Model diagnosis

**Figure 4:**
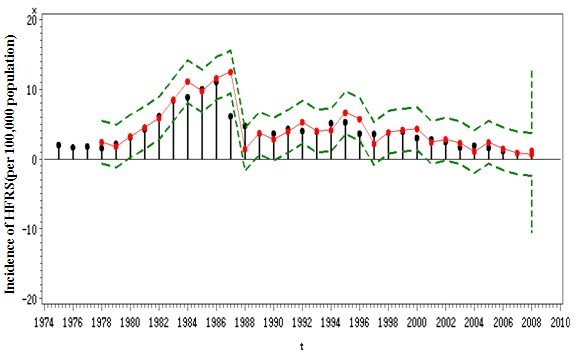
**Black dots: observed values of HFRS incidence, for the period 1975-2008**. Red solid line: ARIMA(0,3,1) model's fitted curve(1978-2008). Red dots: ARIMA(0,3,1) model's fitted values(1978-2008). Dashed lines: 95% confidence intervals.

The parameter estimates for the optimum ARIMA(0,3,1) model are shown in Table 2. The model's fitted  (1975-2008)and predicted values (2009-2011) are presented in Figure 4. The MAPE value was 12.20%. The forecast values of the years 2009, 2010, and 2011 were 0.69, 0.86, and 1.21 per 100,000 population, respectively.

**Table 2:** Parameter for the final ARIMA(0,3,1) model

Parameter	Coefficient	Standard error	*t *statistic	*P *value
MA1	0.9675	0.0599	16.14	< 0.0001

## Discussion

Time series analysis of surveillance data on incidence of various infections is very helpful in developing hypotheses to explain and anticipate the dynamics of the observed phenomena and subsequently in the establishment of a quality control system and reallocation of resources [22]. ARIMA model is one of the most widely used time-series forecasting techniques because of its structured modeling basis and acceptable forecasting performance [23]. In this paper, we applied an ARIMA(p, d, q) model to analyze the surveillance data of HFRS in China. Disease monitoring by public health department entails ongoing data collecting, processing, and updating. However, the national level China CDC is the appropriate level of organization for the implementation of an ARIMA predictive model, because reported data is continually received and updated. We found that model predictions are further improved by the assured availability of the Health Department data. In this study, we have obtained an ARIMA model that closely fits HFRS incidence in China. The autoregression and moving average parameters of our model imply the incidence of HFRS in a month can be estimated by the residual occurring one month prior. According to the results above, the conducted model is reliable with a high validity. Once a satisfactory model has been obtained, it can be used to forecast expected numbers of cases for a given number of future time intervals [24]. Thus, the fitted ARIMA(0,3,1) model can be used to predict the next three years' HFRS incidence in China. The forecast results suggest that the HFRS incidence in China will experience a slight growth in the next three years (2009-2011). A rise in the number of HFRS incidence may also result from an increase in the number and size of natural foci [25], climate change, especially the increase of mean temperature [26,27]. Therefore, knowledge of HFRS forecasts is necessary to prompt health departments to strengthen surveillance systems and reallocate resources in anticipation of increasing HFRS incidence.

Several studies have used ARIMA model to fit and predict changing trends in infectious disease. Luz et al applied an ARIMA(2,0,0)×(1,0,0)_12 _model to predict dengue incidence in Rio de Janeiro [18] and found that ARIMA models were useful tools for monitoring dengue incidence. Earnest et al indicated that ARIMA models provided useful tools for administrators and clinicians in planning for real-time bed capacity during infectious diseases outbreaks such as SARS [28]. Li et al have applied an ARIMA model to monthly incidence of HFRS in Linyi City, China to predict HFRS incidence, and found that the ARIMA model could be used to predict HFRS incidence with high predictive precision in the short-term [29]. In the present study, we further confirmed the consensus that ARIMA model is a useful tool in monitoring and predicting changing trends in infectious diseases.

To the best of our knowledge, this is the first study to apply ARIMA model to fit the HFRS incidence in China with as many as 34 observations at year level. Some previous studies [30,31] in China also used ARIMA model to fit and forecast HFRS incidence of some regions, but they had the same problem that the number of observations was not enough, which led to the instability of their forecast results. In order to conduct a stable and effective ARIMA model, we have to collect at least 30 observations [32]. Thus, parameter estimates of the fitted model would be more robust. The longer the series, the better; however, the series should not extend so far into the past as to include periods during which a different case definition was applied or in which any other reporting artifact resulted in a mean number of cases per interval that differs from the mean of recent intervals. As mentioned above, for adequate ARIMA modeling, a time series should be stationary with respect to mean and variance. If the mean increases or decreases over time, or if the variance does, the series may need to be transformed to make it stationary, before being modeled. Otherwise, the prediction effect of the model will be poor.

In order to improve the model, updating the forecasts is very important. A model without seasonal terms will need to be updated frequently. Confidence intervals that widen rapidly as time increase from the starting point of the forecasts also indicate a model that needs frequent updating. Generally speaking, there are two ways to implement the updating. The model can be reapplied to the original series with extra observations added at the end to give forecasts based on a later starting point. Alternatively, a new model can be fitted to the longer series. This is probably preferable, since fitting a model is quick, especially when the old model is used as a guide, and it makes better use of the additional observations.

Some limitations of this study also need to be taken into account when interpreting the results. In this study, the interval of HFRS incidence is one year, so we could not analyze its seasonal characteristic. In further study, we would use monthly data to predict HFRS incidence in order to get seasonal pattern and higher predictive precision. In addition, the data are from a passive surveillance system, the possible biases in disease reporting and potential underreporting of HFRS cases might influence the precision of our analysis.

## Conclusion

There is an urgent need for monitoring and predicting HFRS incidence to reduce the substantial morbidity and mortality caused by this disease [33]. ARIMA models applied to historical HFRS incidence data are an important tool for HFRS surveillance. Accurate forecasting of the incidence of HFRS is possible. Our modeling approach can be used to monitor and predict HFRS incidence in China. The ARIMA model could be used to optimize HFRS prevention by providing estimates on HFRS incidence trends in China.

## Competing interests

The authors declare that they have no competing interests.

## Authors' contributions

QL, XL, BJ and WY conceived the study, undertook statistical analysis and drafted the manuscript. XL and BJ assisted with data collection and statistical analysis. All authors contributed to the writing of the manuscript and approved the submitted version of the manuscript.

## Pre-publication history

The pre-publication history for this paper can be accessed here:

http://www.biomedcentral.com/1471-2334/11/218/prepub
